# Molecular and Cellular Mechanisms Driving Cardiovascular Disease in Hutchinson-Gilford Progeria Syndrome: Lessons Learned from Animal Models

**DOI:** 10.3390/cells10051157

**Published:** 2021-05-11

**Authors:** Ignacio Benedicto, Beatriz Dorado, Vicente Andrés

**Affiliations:** 1 Centro Nacional de Investigaciones Cardiovasculares (CNIC), 28029 Madrid, Spain; beatrizjulia.dorado@cnic.es; 2 CIBER en Enfermedades Cardiovasculares (CIBER-CV), Spain

**Keywords:** Hutchinson-Gilford progeria syndrome (HGPS), progerin, cardiovascular disease, atherosclerosis, endothelial dysfunction, vascular smooth muscle cell, vascular calcification, extracellular matrix, fibrosis, vessel stiffening, progeroid animal models

## Abstract

Hutchinson-Gilford progeria syndrome (HGPS) is a rare genetic disease that recapitulates many symptoms of physiological aging and precipitates death. Patients develop severe vascular alterations, mainly massive vascular smooth muscle cell loss, vessel stiffening, calcification, fibrosis, and generalized atherosclerosis, as well as electrical, structural, and functional anomalies in the heart. As a result, most HGPS patients die of myocardial infarction, heart failure, or stroke typically during the first or second decade of life. No cure exists for HGPS, and therefore it is of the utmost importance to define the mechanisms that control disease progression in order to develop new treatments to improve the life quality of patients and extend their lifespan. Since the discovery of the HGPS-causing mutation, several animal models have been generated to study multiple aspects of the syndrome and to analyze the contribution of different cell types to the acquisition of the HGPS-associated cardiovascular phenotype. This review discusses current knowledge about cardiovascular features in HGPS patients and animal models and the molecular and cellular mechanisms through which progerin causes cardiovascular disease.

## 1. Hutchinson-Gilford Progeria Syndrome (HGPS)

HGPS (also termed progeria) is a rare genetic disorder (estimated prevalence of 1 in 18–20 million people) caused by a mutation in the *LMNA* gene that provokes accelerated aging and premature death [1,2,3]. In mammals, *LMNA* normally encodes two major alternatively spliced A-type lamin variants, lamin A and lamin C, which are main components of the nuclear lamina. Post-translational modifications of prelamin A that yield mature lamin A include C-terminal farnesylation and carboxymethylation and subsequent cleavage of the modified C-terminus by the zinc metalloprotease ZMPSTE24. “Classical” progeria is caused by a heterozygous de novo c.1824C>T (p.G608G) point mutation in the *LMNA* gene. This creates an aberrant splice site in exon 11 that deletes 150 nucleotides, resulting in the synthesis of a truncated prelamin A variant called progerin that lacks the cleavage site needed to remove the farnesylated and carboxymethylated C-terminus [4]. Progerin is expressed in most differentiated cells and induces multiple cellular and physiological anomalies, including defects in nuclear morphology, chromatin disorganization, elevated DNA damage, impaired stem cell maintenance and differentiation, metabolic alterations, autophagy deregulation, and systemic inflammation [5].

HGPS patients appear normal at birth, but start to develop a premature and accelerated aging phenotype between 1 and 2 years of age. Typical features include failure to thrive, alopecia, loss of subcutaneous fat, skin alterations, tooth and bone abnormalities, muscle weakness, and progressive joint contractures. However, the most clinically relevant feature of HGPS is the development of generalized atherosclerosis and cardiac dysfunction, which ultimately lead to death mainly due to myocardial infarction, heart failure, or stroke at an average age of 14.6 years [4,6]. The following section describes the HGPS-associated cardiovascular phenotype in detail.

## 2. The Cardiovascular Phenotype in HGPS Patients: A Historical Perspective

Cardiovascular disease (CVD) is the main cause of death in HPGS, and it is therefore crucial to decipher the underlying mechanisms in order to design effective therapies. Because HGPS patients typically lack many of the classical cardiovascular risk factors such as hypercholesterolemia, high C-reactive protein, obesity, and smoking, the study of the mechanisms underlying CVD in HGPS also presents an opportunity to understand CVD in non-HGPS individuals in relative isolation from confounding risk factors. A robust characterization of the HGPS cardiovascular phenotype is also critical for developing clinical standards of care and objective cardiovascular readouts of therapeutic efficacy in HGPS clinical trials.

The extreme rarity of HGPS hinders systematic cardiovascular examination of patients, because the very few HGPS patients are spread around the world, may be diagnosed at different ages, and have very different access to primary and specialized care. However, information gathered over the years from individual HGPS case reports, autopsies, and a few clinical trials has helped to define a fairly consistent cardiovascular phenotype. The invaluable HGPS literature prior to the discovery of the genetic cause of the disease (from 1886 to 2003) included patients diagnosed by their characteristic physical appearance, including the so called “old-mannish” look, with a disproportionally big head, baldness, prominent eyes, thin lips, pointed nose, wrinkled and thin skin, and extreme thinness [7,8]. This allowed the differentiation of HGPS patients from patients with other progeroid-like diseases. Since 2003, HGPS clinical reports include only individuals with confirmed genetic tests, which have identified the classical *LMNA* c.1824C>T mutation in most patients (https://www.progeriaresearch.org/wp-content/uploads/2021/04/FINAL1-PRF-By-the-Numbers_-March-2021.pdf, accessed on March 2021). These recent reports support the previously described HGPS phenotype and have broadened our understanding of HGPS cardiovascular pathology through the use of modern clinical techniques for assessing vasculopathy, such as the measurement of vessel wall echodensity, ankle-brachial index, and pulse wave velocity (PWV). Here, we present a chronological overview of the cardiovascular characterization of HGPS patients that gathers together information from the landmark literature on the clinical definition of HGPS pathology.

### 2.1. Atherosclerosis and Cardiovascular Calcification

The first thorough anatomical description of a child with remarkable signs of premature aging was reported in 1886 by J. Hutchinson [9]. A decade later, H. Gilford found that, with no family relationship, Hutchinson’s patient shared a striking physical resemblance to one of his own patients, who looked like an old man, had heart problems, and died at the age of 17 [10]. In the detailed autopsy of this second patient, Gilford noted extensive atheromatous degeneration of the mitral and aortic orifices, obliteration of the lumen of the coronary arteries, soft atheromatous patches on the convex surface of the aortic arch, patches of calcareous material on the concave surface, and extensive atheroma with collections of organized fibrin in the aortic branches [10].

In 1972, F. L. DeBusk reported four new cases of HGPS and compiled the first thorough list of progeria-like cases from the worldwide literature. In one of his patients, he found enlargement of the right ventricle and a calcified heart valve, and in another he found obstruction of the proximal left coronary artery [7]. Examination of autopsies revealed that most patients with typical HGPS die of congestive heart failure or acute myocardial infarction due to primary underlying coronary atherosclerosis, and that all patients had varying degrees of generalized atherosclerosis, mainly affecting the large arteries [7].

In a later review of 12 autopsies, P. B. Baker and colleagues found that cardiac complications in progeria patients were rarely due to cardiomyopathy (interstitial fibrosis in the myocardium without severe coronary artery disease); rather, most cardiac manifestations were due to atherosclerosis-related coronary artery narrowing or occlusion [11]. Atherosclerosis and calcification of aortic and mitral valves were also common, and all cases featured atherosclerotic plaques in the aorta, ranging from small fatty streaks to complicated calcified lesions. In addition, Baker et al. reported the case of a patient with significant thickening of the intima and media in small cardiac intramural arteries accompanied by diffuse interstitial fibrosis, suggesting small artery disease as a possible cause of cardiac ischemia [11].

### 2.2. Vascular Smooth Muscle Cell (VSMC) Loss and Vascular Fibrosis

In 2001, W. E. Stehbens and colleagues reported an unusual histological finding upon reviewing two new cases of HGPS patients who had died of myocardial infarction [12]. Both patients had severe depletion of VSMCs in the aortic media. The VSMCs were replaced by collagen fibrils, and their depletion correlated with the presence of atherosclerosis and hemodynamic stress around branch sites. The author concluded that the VSMC debris in the fibrosed medial layer indicated muscle degeneration rather than muscle atrophy, and that increased arterial fibrosis may reduce the viscoelastic properties of the wall and contribute to increased blood pressure.

A further analysis in 2006 by R. C. M. Hennekam reported 10 new cases of HGPS and reviewed 132 cases from the literature [8]. The report covered the range from individual phenotypic features to common symptoms shared by all patients. None of the patients showed signs of a cardiovascular phenotype until around 6 to 8 years of age, when it began to manifest as shortness of breath with exertion and easy fatigability. Other common cardiovascular features included an increase in heart rate and blood pressure with age, heart enlargement and impaired coronary function attributable to an enlarged left ventricle, hypertrophy of myocardial cells and cardiac interstitial fibrosis, angina pectoris appearing up to five years before death, extensive VSMC loss and thickening of the coronary arteries with atheroma plaques (with and without calcification), and thickening of the aortic leaflets, which were often calcified. In addition, half the patients had mitral valve defects, and the aorta had a very variable appearance, from almost normal to severely atherosclerotic. Vascular problems in these patients often extended to the brain, with silent or symptomatic strokes and cerebral infarction frequently reported.

The first prospective clinical characterization of HGPS patients was reported in 2008 by M. A. Merideth et al. [13]. These authors followed 15 HGPS patients aged between 1 and 17 years over 16 months. Most of the patients had elevated platelet counts, a prolonged prothrombin time, and elevated serum phosphorus. Another frequent feature was the development of age-dependent impaired vascular function, including elevated blood pressure, increased arterial augmentation rate, and reduced ankle-brachial index, together with adventitial thickening. Less frequent abnormalities were thickened aortic valves with regurgitation, left ventricular hypertrophy, pulmonary hypertension, stenotic lesions in the cerebral and carotid arteries, and electrocardiographic alterations that were not associated with ischemia [13].

In 2010, M. Olive et al. published the first structural and immunohistological comparison of cardiovascular tissues between HGPS and non-HGPS individuals, examining two HGPS patients who died of myocardial infarction and a small non-HGPS cohort composed of 29 individuals with or without CVD with ages ranging from 1 month to 97 years [14]. Atherosclerosis in HGPS patients and in normally aged individuals shared several features, such as severe stenosis, extensive arterial calcification, and the presence of a range of early- to late-stage atherosclerotic lesions displaying calcification, inflammation, and evidence of erosion or rupture. However, HGPS lesions tended to have smaller atheromatous cores, very large regions of calcification, and thick fibrosis and were categorized predominantly as fibro-calcific lesions and less frequently as fibro-atheromas. Moreover, compared with geriatric vessels, all HGPS vessels (arteries and veins) showed prominent adventitial fibrosis. This study also revealed that progerin is expressed not only in the vessels of HGPS patients, but also in a small subset of cells in the coronary arteries of non-HGPS individuals, with progerin levels increasing with age [14]. Additional studies have confirmed low progerin expression in several human cell types and tissues [15,16,17,18,19,20,21,22,23,24,25,26,27,28], but its functional relevance remains to be determined.

### 2.3. Vascular Stiffening: A Useful Readout for Clinical Trials

Researchers have also investigated whether end-stage cardiovascular events in HGPS are related to progressive impairment of vascular compliance. In a prospective single-center study, M. Gerhard-Herman et al. enrolled 26 HGPS patients and 62 age- and gender-matched healthy children [29]. All the HGPS patients exhibited vascular stiffness, evidenced by markedly elevated carotid-femoral PWV values comparable to those in adults older than 60 years. This finding was later corroborated in another study [30]. Other HGPS patient characteristics included higher carotid echobrightness in the intima-media, near the adventitia and deep adventitia, indicating increased arterial wall density and suggesting accumulation of thick collagen fibrils, as well as elevated internal carotid artery mean flow velocity and abnormal ankle-brachial indices, indicating occlusive stenosis and peripheral vascular disease, respectively [29]. HGPS was therefore classified as a disease of vascular stiffening in the setting of gradual vascular stenosis, similar to CVD in normal aging. Subsequently, PWV and arterial wall echodensity became key cardiovascular readouts of therapeutic efficacy in HGPS clinical trials [31,32,33]. The first HPGS clinical trial showed improved vascular stiffness upon treatment with the farnesyltransferase inhibitor lonafarnib, from pre-therapy PWV values typical of people aged 60–69 years to end-of-therapy PWV values corresponding to 40–49-year-old individuals [31]. Moreover, lonafarnib treatment reduced the echobrightness of the carotid intima-media and near/deep adventitia. These findings thus suggested that lonafarnib therapy had the potential to reduce fatal and not-fatal HGPS cardiovascular events and strokes. A second clinical trial showed that triple therapy with lonafarnib plus the statin pravastatin and the bisphosphonate zoledronic acid produced benefits in bone mineral density but did not improve vascular stiffness or structure more than lonafarnib alone [32]. Instead, the triple therapy resulted in increased development of carotid and femoral arterial plaques and extraskeletal calcifications, thus discouraging recommendation of its use for the clinical treatment of HGPS [32]. The results from both clinical trials indicated that single lonafarnib therapy can significantly lower mortality rate [33], and in light of these studies, in November 2020 lonafarnib (marketed as Zokinvy) became the first U.S. Food and Drug Administration-approved drug for HGPS [34].

### 2.4. Cardiac Dysfunction

Clinical evaluation of potential treatments for HGPS also requires the definition of appropriate cardiac endpoints. Several electrocardiographic defects have been reported in HGPS patients [13,30,35], and one defect type frequently found as patients age is cardiac repolarization anomalies [35,36]. Regarding cardiac anatomy and function, Prakash et al. recently reported that the most frequent echocardiographic abnormality showing increased prevalence with age in HGPS patients is left ventricular (LV) diastolic dysfunction [30]. The authors speculated that LV dysfunction might be a consequence of myocardial interstitial fibrosis and endocardial thickening, which was observed in two previous case reports [8,14]. Other cardiac alterations, such as LV hypertrophy and aortic valve calcification and dysfunction, were less common and more frequently seen during the second decade of life [30]. In line with previous observations in the carotid artery [29], 70% of HGPS patients had unusually high echobrightness in the aortic root wall, and these patients were more likely to present with diastolic LV dysfunction [30]. Interestingly, increased echobrightness was not associated with age, suggesting that extracellular matrix (ECM) remodeling may be an early alteration potentially underlying the development of the cardiac phenotype. Another recent study [37] applied three-dimensional echocardiography (speckled tracking imaging) to analyze seven HGPS patients (five children ≤ 8 years of age and two children in their teens), 21 aged-matched healthy children, and 14 older healthy volunteers (mean age: 65.7 ± 7.5). Despite the fact that most HGPS patients were in their first decade of life, the authors found one case of diastolic dysfunction and another of aortic valve calcification, severe aortic stenosis, and LV hypertrophy, in agreement with some of the findings reported by Prakash et al. [30].

The data accumulated over several decades indicates that the HGPS cardiovascular phenotype is defined by generalized atherosclerosis with a wide spectrum of early- to late-stage atherosclerotic plaques, prominent VSMC loss, vascular stiffening, calcification and fibrosis, cardiac repolarization abnormalities, LV diastolic dysfunction, and cardiac valve disease, all of which probably contribute to the death of HGPS patients from myocardial infarction, stroke, or heart failure. However, many fundamental questions remain unanswered, and the natural history of CVD leading to these death-causing pathologies is ill defined. Moreover, it remains to be determined whether LV diastolic dysfunction and the altered electrocardiographic activity commonly observed in HGPS patients are early cardiovascular complications or are secondary to other chronic pathologies that precede their onset, such as long-term vascular stiffening and calcification, fibrosis, and ischemia. It is therefore essential to investigate the natural history of HGPS-associated CVD and its underlying mechanisms in order to provide clinicians with the scientific support they need to fight disease progression earlier and more efficiently. The discovery of the genetic mutation causing classical HGPS in 2003 [2,3] triggered the generation of a number of HGPS experimental models that play a pivotal role in the investigation of progerin-induced alterations and the testing of new therapeutic approaches. In the next section we describe the major HGPS-like animal models, with a special emphasis on those that recapitulate aspects of HGPS-associated CVD.

## 3. The Cardiovascular Phenotype in Animal Models of HGPS

The use of animal models in HGPS research is particularly important given the low number of HGPS patients worldwide, which makes it very challenging to perform longitudinal studies and clinical trials and reduces the availability of human samples for ex vivo analysis. Over the past two decades, several HGPS animal models have been developed to study different aspects of the disease (Table 1). In 2002, two laboratories developed the *Zmpste24^−/−^* mouse model, which lacks the metalloprotease ZMPSTE24 responsible for cleaving the farnesylated and carboxymethylated C-terminus of prelamin A. Homozygous *Zmpste24^−/−^* mice accumulate irreversibly modified prelamin A, lack mature lamin A, and develop a bona fide progeroid phenotype, including muscle and bone defects, alopecia, lipodystrophy, cardiac alterations, and reduced lifespan [35,38,39]. However, with the exception of perivascular fibrosis in coronary arteries, no major vascular alterations have been reported in *Zmpste24^−/−^* mice. This situation thus left a need for mouse models that more accurately resemble HGPS pathology. The first progerin-expressing HGPS murine model was the *Lmna^HG^* mouse, generated in 2005 [40,41]. Heterozygous *Lmna^HG/+^* mice express progerin, lamin A, and lamin C, whereas homozygous *Lmna^HG/HG^* mice only express progerin. Both *Lmna^HG/+^* and *Lmna^HG/HG^* mice develop HGPS-like disease, including slow growth, bone abnormalities, fat loss, and premature death. However, these mice have not been reported to develop vascular abnormalities and therefore are not suitable for the study of HGPS-associated vascular pathology, the most clinically relevant feature of the disease.

The G608G BAC mouse model, developed in 2006, expresses human lamin A, lamin C, and progerin together with endogenous mouse lamin A and lamin C and was the first HGPS animal model to recapitulate some aspects of the HGPS-associated vascular phenotype [42]. Heterozygous G608G BAC animals have a normal lifespan and do not present consistent abnormalities in bone, muscle, skin, or heart [42], whereas homozygous mice show musculoskeletal and skin anomalies, lack subcutaneous fat, and die prematurely [43,63]. However, both heterozygous and homozygous G608G BAC mice develop a marked vascular phenotype in the aorta, carotid artery, and iliac artery, including progressive loss of VSMCs, thickening of the adventitia and medial layer, and vessel calcification in older animals. Histological analysis and transmission electron microscopy studies also revealed medial accumulation of proteoglycans and disorganized collagen fibrils, together with alterations to elastic fibers. These structural abnormalities are accompanied by defective arterial responses to the VSMC-dependent vasodilator sodium nitroprusside [42]. G608G BAC mice therefore constitute a very valuable tool for studying several features of the HGPS-associated vascular phenotype and testing new therapeutic approaches. Indeed, work in homozygous G608G BAC mice recently demonstrated that treatment with *LMNA*-targeted antisense oligonucleotides reduces progerin levels and increases longevity [44,64], and this was accompanied by amelioration of VSMC depletion and adventitial thickening in one of the studies [44]. In another study, partial correction of the HGPS-associated mutation *LMNA* c.1824C>T by in vivo systemic base editing in homozygous G608G BAC mice led to the total prevention of VSMC loss and adventitial thickening and a striking 2.4-fold increase in lifespan [43]. Potential limitations of the G608G BAC model are the unknown effects of overexpressing human progerin in the context of endogenous mouse lamin A and lamin C expression, and the presence of the human genes *UBQLN4*, *MAPBPIP*, and *RAB25* in the *LMNA*-containing bacterial artificial chromosome used to generate G608G BAC mice [42].

The *Lmna^G609G^* HGPS mouse model, generated in 2011 by the López-Otín laboratory, was the first mouse model to express progerin due to aberrant splicing of the endogenous *Lmna* gene, resulting from the introduction of the 1827C>T (p.G609G) mutation, which is equivalent to the human mutation found in HGPS patients [45]. Using this model, these authors provided the first in vivo evidence that antisense morpholino-based therapy to prevent the pathogenic *Lmna* splicing might be viable for HGPS. Homozygous *Lmna^G609G/G609G^* mice express progerin, lamin C, and residual levels of lamin A and show many HGPS features, including failure to thrive, bone defects, loss of fat deposits, bradycardia, prolonged QRS waves (indicating altered heart ventricular depolarization), and premature death [45,49,52]. *Lmna^G609G/G609G^* cardiomyocytes have structural, conduction, and excitation-contraction coupling defects, all of which can be partially corrected by chronic treatment with the microtubule-stabilizing drug paclitaxel [49]. Vascular alterations include VSMC depletion in the medial layer of the aorta and other arteries, collagen and proteoglycan accumulation in the aortic media, reduced elastin fiber undulations, and increased vessel stiffness assessed by wire and pressure myography [45,51,55,56,65]. Aortic VSMC loss and adventitial fibrosis were also features of a separate *Lmna^G609G/G609G^* mouse model developed independently in 2016 by the Fong laboratory [54]. Compared with age-matched wild-type animals, the aortic and carotid medial layers of *Lmna^G609G/G609G^* mice show marked accumulation of the collagen-crosslinking enzyme lysyl oxidase (LOX), which probably contributes to progerin-induced vessel stiffening [55]. Heterozygous *Lmna^G609G/+^* mice also die prematurely, but their average lifespan (34 weeks) is considerably longer than that of homozygous *Lmna^G609G/G609G^* mice (15 weeks) [45]. The longer lifespan of the heterozygotes allows the study of vascular alterations that take longer to manifest, such as vessel calcification, which is prominent in the aortic arch and thoracic aorta of ≈30-week-old *Lmna^G609G/+^* mice [46,47,48].

Although the G608G BAC and *Lmna^G609G^* mouse models recapitulate many of the vascular alterations found in HGPS patients, they do not develop atherosclerosis, one of the main features in HGPS patients. This is most likely due to very low levels of pro-atherogenic lipoproteins in mice, which confers natural resistance to developing atherosclerosis [66]. To overcome this limitation, the *Apoe^−/−^Lmna^G609G/G609G^* mouse model was generated [53]. These mice harbor the progerin-producing *Lmna* mutation and are engineered to lack apolipoprotein E, a genetic manipulation that has been extensively demonstrated to induce atherosclerosis in rodents [66]. Similar to *Lmna^G609G/G609G^* mice, *Apoe^−/−^Lmna^G609G/G609G^* mice show postnatal growth defects, VSMC loss in the aortic arch, cardiac electrical anomalies, and reduced lifespan compared to *Apoe^−/−^Lmna^+/+^* controls; however, *Apoe^−/−^Lmna^G609G/G609G^* mice also show adventitial thickening, accumulate fluorescently-labeled human low-density lipoproteins (LDLs) in the medial layer of atheroma-free aortic regions, and develop aggravated aortic atherosclerosis without evidencing higher serum cholesterol or LDL than *Apoe^−/−^Lmna^+/+^* mice. *Apoe^−/−^Lmna^G609G/G609G^* mice also showed signs of plaque vulnerability in the aortic root, accompanied by fibrosis and inflammation in the adjacent cardiac tissue, and some showed evidence of myocardial infarction at necropsy [53]. The *Apoe^−/−^Lmna^G609G/G609G^* mouse is thus the first HGPS animal model to allow the study of HGPS-associated atherosclerosis.

The same laboratory subsequently developed another atheroprone HGPS model, the *Ldlr^−/−^Lmna^G609G/G609G^* mouse [59], which harbors the progerin-causing *Lmna* mutation and ablation of the LDL receptor gene (*Ldlr*), another well-established strategy for inducing atherosclerosis in mice [66]. The *Ldlr^−/−^Lmna^G609G/G609G^* mouse phenotype is very similar to that of *Apoe^−/−^Lmna^G609G/G609G^* mice, but with less pronounced adventitial thickening and less fibrosis and inflammation in the tissue neighboring the aortic root. The similar vascular phenotypes of these models indicates that the observed alterations are due to the effects of progerin expression, and not to the specific deletion of *Apoe* or *Ldlr*. A potential limitation of both the *Apoe^−/−^Lmna^G609G/G609G^* and *Ldlr^−/−^Lmna^G609G/G609G^* mouse models is that the animals develop severe hypercholesterolemia and hypertriglyceridemia, conditions that are generally absent in HGPS patients [13,67], who also show reduced HDL cholesterol levels with age [67]. It is therefore possible that the altered lipid profile might to some extent modulate the vascular anomalies induced by progerin expression.

Studies using G608G BAC mice and the *Lmna^G609G/G609G^* model and its atheroprone variants have made a significant contribution to the assessment of the HGPS-associated vascular phenotype. The most suitable model for analysis will depend on the vascular alteration of interest. For example, G608G BAC and homozygous *Lmna^G609G/G609G^* mice can be used to decipher the mechanisms of VSMC loss, medial collagen accumulation, increased arterial stiffness, and other HGPS-related vascular anomalies in the absence of hyperlipidemia. In contrast, heterozygous G608G BAC and *Lmna^G609G/+^* mice are useful tools for analyzing progerin-induced vessel calcification, whereas *Apoe^−/−^Lmna^G609G/G609G^* and *Ldlr^−/−^Lmna^G609G/G609G^* mice are suitable models for the study of HGPS-associated atherosclerosis.

Research with mouse models has significantly advanced knowledge about HGPS-associated pathology, including CVD, and has paved the way to the development of therapies with the potential to ameliorate or even cure HGPS [43,44,64,68,69,70]. However, therapies that are effective in HGPS-like mouse models have yielded only modest benefit in HGPS patients [31,32,33]. In this context, decision-making about scheduling candidate treatments for clinical trials [71] would be greatly facilitated by prior validation in large animal models more closely resembling human physiology. Large animal models also allow the use of the same devices employed to study cardiovascular alterations in human patients, which can help standardize surgical models or medical protocols before moving into clinical practice. The first large animal model of HGPS was generated in 2019 in Yucatan minipigs. The modified pigs bear a heterozygous c.1824C>T mutation in the *LMNA* gene and show severe growth retardation, lipodystrophy, skin and bone alterations, and premature death [61]. Importantly, they also show cardiovascular alterations seen in HGPS patients, including LV diastolic dysfunction, altered cardiac electrical activity, and VSMC depletion. Moreover, the hearts of HGPS-like minipigs develop interstitial fibrosis and defective myocardial perfusion, together with degenerated coronary arteries and increased collagen content in the medial and adventitial layers of cardiac arterioles. The human-like size of the HGPS minipig model allowed continuous in vivo monitoring of cardiac electrical activity with subcutaneously implanted loop recorders, which revealed severe pre-mortem cardiac conduction abnormalities in some animals. These included advanced third-degree atrio-ventricular block at the moment of death and complete atrio-ventricular block preceded either by short-duration polymorphic ventricular tachycardia or by ST-segment elevation, which was not present 29 h before death, suggesting a coronary ischemic event potentially related to microvascular dysfunction and vascular fibrosis. In 2020, the same c.1824C>T mutation was introduced into the *LMNA* gene of cynomolgus monkeys to generate the first non-human primate HGPS model [62]. Although lifespan studies were not reported, progeroid monkeys recapitulated typical HGPS phenotypes, including growth retardation, bone and joint alterations, skin problems, alopecia, and loss of subcutaneous fat. HGPS-like monkeys also show an increase in vascular wall collagen deposition and signs of intimal hyperplasia in the aorta, although these alterations were only reported in monkeys homozygous for the *LMNA* mutation.

Over almost two decades, the generation of multiple HGPS animal models has enabled a thorough characterization of the HGPS-associated cardiovascular phenotype and the discovery of mechanisms that mediate vascular damage. In the next section, we examine progerin-induced alterations in VSMCs and endothelial cells (ECs), major vessel-wall constituents that play key roles in the development and complications of atherosclerotic CVD. We also discuss the potential role of progerin expression in these cell types in the acquisition of HGPS-associated cardiovascular phenotype. Multiple in vitro studies have made important contributions to the characterization of progerin-induced alterations in VSMCs and ECs [72,73,74,75,76,77,78,79,80,81,82]; however, in the interest of conciseness, here we focus mainly on in vivo and ex vivo data obtained from HGPS animal models.

## 4. Role of VSMCs and ECs in HGPS-Associated Cardiovascular Dysfunction

Progerin expression has been detected in VSMCs and ECs in HGPS patients [14,16]. VSMCs are a key structural and functional element of the vessel wall, indispensable for maintaining vascular tone and regulating blood pressure thanks to their contractile properties [83,84]. In pathological scenarios such as atherosclerosis, VSMCs progressively acquire a defective contractile phenotype and undergo transdifferentiation into a wide range of different cell types, including macrophage-like cells, adipocyte-like cells, cells with an osteochondrogenic phenotype, and ECM-producing myofibroblast-like cells. Endothelial dysfunction is a major determinant of the initiation and progression of atherosclerosis [85]. Increased endothelial permeability facilitates subendothelial lipid deposition and leukocyte extravasation into the intima, which drives atherosclerotic plaque formation [86]. Moreover, there is strong evidence that an abnormally stiff ECM perturbs intercellular endothelial junctions [87] and can promote paracellular leukocyte transmigration [88,89]. Interestingly, reducing arterial ECM stiffness with the LOX inhibitor β-aminopropionitrile (BAPN) attenuates atherosclerosis in *Apoe* knockout mice [90]. ECM stiffening in HGPS could therefore contribute to atherosclerosis development by directly altering endothelial phenotype and function. Because the individual functions of VSMCs and ECs are dependent on proper communication between these cell types [91,92], slight perturbations to this signaling circuit can generate a feedback loop with detrimental effects on vessel structure and function. Understanding progerin-induced structural, functional, and molecular alterations to the vascular ECM, VSMCs, and ECs and their interactions is therefore crucial for the development of new vascular-targeted therapies for HGPS.

### 4.1. VSMCs in HGPS Mouse Models

HGPS patients and animal models both show prominent VSMC depletion, which may be involved in the development of life-threatening cardiovascular complications associated with the disease. To date, the most widely used experimental system for the study of progerin-induced VSMC alterations in vivo is the *Lmna^G609G^* model. Homozygous *Lmna^G609G/G609G^* mice show no obvious aortic structural alterations at 8 weeks of age [55,65], but VSMC depletion is evident in the aortic arch at 12–14 weeks [45,51] and also in the thoracic aorta at 17 weeks [65]. These observations reinforce the idea that HGPS-associated VSMC loss is progressive and that the aortic arch may be especially sensitive to progerin-induced vascular damage, due to environmental factors such as turbulent blood flow and other mechanical cues characteristic of this aortic segment [93]. Consistent with this idea, VSMC depletion in the aortic arch of *Lmna^G609G/G609G^* mice is more severe on the inner than the outer curvature [65], correlating directly with exposure to more pronounced blood flow disturbance. The thoracic aorta of ≈14-week-old *Lmna^G609G/G609G^* mice shows no evidence of VSMC loss but does show reduced numbers of smooth muscle fibers (measured by hematoxylin staining) and increased deposition of medial collagen [51]. Moreover, vessel contraction in response to phenylephrine and potassium chloride is impaired in thoracic aorta segments from these progeroid mice [50,56], and this effect is not rescued by collagen disruption after collagenase treatment [50]. In line with this evidence of progressive damage, dysfunction, and dedifferentiation of VSMCs in *Lmna^G609G/G609G^* mice, atheroprone *Apoe^−/−^Lmna^G609G/G609G^* mice show VSMC depletion in the aortic arch at 16 weeks of age but not at 8 weeks [53]. An RNA sequencing analysis to identify potential mechanisms causing VSMC loss explored gene expression differences between aortas from 8-week-old *Apoe^−/−^Lmna^G609G/G609G^* mice and age-matched control *Apoe^−/−^Lmna^+/+^* animals [58]. To enrich for VSMC-expressed genes, the adventitia was removed before RNA extraction. The most significant differences between control and progerin-expressing aortas were in genes related to fibrosis, oxidative stress, and, interestingly, the endoplasmic reticulum (ER) stress response and the ER stress-related unfolded protein response. Treatment of *Apoe^−/−^Lmna^G609G/G609G^* mice with tauroursodeoxycholic acid (TUDCA), a chemical chaperone that inhibits ER stress, significantly reduced medial VSMC loss and atherosclerosis burden [58].

Additional genetic and pharmacological strategies have proved successful in reducing or preventing VSMC loss in HGPS-like mice. These strategies include deletion of the matrix metalloprotease 13 (*Mmp13*) gene, the use of the matrix metalloprotease inhibitor batimastat [94], and the blockade of interleukin-6 signaling with the neutralizing antibody tocilizumab [95] in *Lmna^G609G/G609G^* mice, suggesting that HGPS-associated VSMC depletion involves ECM remodeling and inflammation. VSMC loss in *Lmna^G609G/G609G^* mice was also prevented by inhibition of N-acetyltransferase 10 with remodelin, and this treatment also ameliorated aortic adventitial thickening and extended lifespan [96]. In G608G BAC mice, VSMC depletion was reduced by treatment with the farnesyltransferase inhibitor tipifarnib [97] and by lowering progerin levels with *LMNA*-targeting antisense oligonucleotides [44]. Another effective means of ameliorating VSMC loss in *Lmna^G609G/G609G^* mice is the inhibition of progerin carboxymethylation by inactivating the isoprenylcysteine carboxylmethyltransferase (*Icmt*) gene, a strategy that also increased lifespan in heterozygous *Lmna^G609G/+^* mice [98]. These findings were complemented by in vitro experiments showing that drug-based ICMT inhibition delayed senescence and stimulated proliferation of late-passage HGPS cells and *Zmpste24*-deficient mouse fibroblasts but had no effect on the proliferation of wild-type human cells or *Zmpste24*-deficient mouse cells lacking *Icmt* [98]. A number of therapeutic strategies have also shown beneficial effects on other HGPS-associated vascular phenotypes related to VSMC dysfunction. In *Lmna^G609G/G609G^* mice, increased vascular stiffness and defective vascular tone were partially prevented by dietary supplementation with sodium nitrite [50,51], and vessel calcification was reduced after pyrophosphate treatment [48]. Interestingly, LOX inhibition with BAPN prevented carotid stiffening and diastolic dysfunction in 8-week-old *Lmna^G609G/G609G^* mice [55], strongly suggesting that ECM remodeling plays a key role in the acquisition of the HGPS-associated cardiovascular phenotype. Moreover, aortic calcification in heterozygous *Lmna^G609G/+^* mice was reduced by ATP-based therapy or dietary supplementation with magnesium, and this outcome was accompanied by a concomitant increase in lifespan [46,47].

VSMC depletion in HGPS patients and animal models is well established; however, it is only recently that attempts have been made to assess the causal role of progerin-expressing VSMCs in the acquisition of HGPS-associated vascular phenotype. In one approach, *SM22αCre* transgenic mice (with Cre activity in embryonic VSMCs and cardiomyocytes) [99] were crossed with *Lmna^LCS/LCS^* mice (harboring the 1827C>T mutation in the endogenous *Lmna* gene after an intronic transcriptional stop signal flanked by LoxP sites) [45]; the resulting *Lmna^LCS/LCS^SM22αCre* mice express progerin predominantly in VSMCs and cardiomyocytes [50,51,53]. Like *Lmna^G609G/G609G^* mice with ubiquitous progerin expression, *Lmna^LCS/LCS^SM22αCre* mice have aortic VSMC loss, increased stiffness, and impaired contraction [50,51,53]. In the context of *Apoe* deletion, *Apoe^−/−^Lmna^LCS/LCS^SM22αCre* mice develop similar vascular alterations to those found in *Apoe^−/−^Lmna^G609G/G609G^* mice, including VSMC depletion, adventitial thickening, and increased atherosclerosis burden [53], all of which are also prevented by treatment with the ER stress-targeting chaperone TUDCA [58]. These results strongly suggest that VSMC-intrinsic alterations play a central role in the development of the HGPS vascular phenotype. However, it is worth noting that unlike 14–16-week-old *Lmna^G609G/G609G^* mice, *SM22αCre*-driven progerin expression in age-matched animals was not sufficient to impair acetylcholine-induced vasorelaxation [50] (see below) or induce cardiac electric alterations in the context of *Apoe* deletion [53]. Therefore, it is likely that full development of the HGPS-associated cardiovascular phenotype requires progerin expression in other cell type(s), in addition to VSMCs and cardiomyocytes. Moreover, although *Lmna^LCS/LCS^SM22αCre* and *Apoe^−/−^Lmna^LCS/LCS^SM22αCre* mice both show VSMC depletion and adventitial thickening, the former have normal lifespan [53]. In line with the normal lifespan of VSMC-depleted non-atherogenic HGPS-like mice, heterozygous G608G BAC mice show aortic VSMC loss but have a normal life expectancy [42]. These observations indicate that, at least in mice, progerin-induced VSMC loss, increased vascular stiffness, and reduced vascular contractility in the absence of a pro-atherogenic environment are not sufficient to shorten lifespan. Moreover, when hyperlipidemia is not present (as is the case with HGPS patients), the development of life-threatening cardiovascular complications may require progerin expression in other cell type(s) in addition to VSMCs. Further studies are needed to fully define the direct and indirect roles of VSMCs in the development of the HGPS vascular phenotype and to determine whether targeting progerin expression or downstream effectors specifically in VSMCs can prevent the development of HGPS-associated vascular alterations.

The cytoskeleton and nuclear lamina are connected through the linker of nucleoskeleton and cytoskeleton (LINC) complex, which mediates physical force transmission between these compartments. Progerin-expressing cells show altered expression and diffusional mobility of LINC nuclear membrane proteins [100,101], and *SM22αCre*-driven disruption of the LINC complex in *Lmna^G609G/G609G^* mice ameliorates aortic VSMC loss and adventitial fibrosis [65]. In addition, progerin expression in cultured mouse VSMCs increases cell death in response to mechanical stress in a LINC complex-dependent manner [65]. In line with these findings, in vitro biomechanical assays have shown that progerin expression induces nuclear stiffening and defective nucleocytoskeletal force transmission [77,82,102,103]. Notably, mechanical strain decreases the viability of progerin-expressing primary skin fibroblasts and increases pro-inflammatory gene expression in progerin-expressing iPSC-derived VSMCs, both generated from HGPS patients [104,105]. These results strongly suggest a direct link between progerin-induced anomalies in LINC-mediated VSMC nuclear mechanosensing and the development of HGPS-related vascular alterations. Nevertheless, it has not been reported whether VSMC-specific disruption of the LINC complex increases the lifespan of *Lmna^G609G/G609G^* mice [65], and further studies are therefore needed to assess the efficacy of VSMC targeting to extend survival in HGPS animal models.

### 4.2. ECs in HGPS Mouse Models

In contrast to the progerin-induced alterations in the medial and adventitial layers—enriched in VSMCs and fibroblasts, respectively—the aortic luminal endothelium of HGPS mice appears to be histologically intact, even in areas with severe VSMC depletion [42]. Moreover, a cohort of 14 HGPS patients showed normal flow-mediated brachial artery dilation, suggesting preserved endothelial reactivity [13]. However, even subtle perturbations in ECs can induce endothelial dysfunction, considered central to the onset and development of atherosclerosis [85]. *Lmna^G609G/G609G^* mice show defective endothelium-dependent aortic dilation in response to acetylcholine, a clear sign of endothelial dysfunction [50,57], prompting interest in progerin-induced endothelial alterations and their potential role in the development of the HGPS-associated cardiovascular phenotype. Recent single-cell RNAseq studies in native, non-cultured mouse lung ECs revealed significant enrichment in transcriptional changes related to the inflammatory response in cells from *Lmna^G609G/G609G^* mice (ubiquitous progerin expression) [57]. Further studies are needed to identify and assess the role of progerin-induced transcriptional alterations in ECs from tissues more closely related to HGPS pathology, such as heart and aorta.

Some recent studies have investigated the effects of endothelium-specific progerin expression on the development of HGPS-associated features. Mice carrying human lamin A minigenes with the HGPS 1824C>T mutation were crossed with transgenic mice expressing a tetracycline-responsive transcriptional activator under the control of the EC-specific *Cdh5* promoter, generating *Prog-Tg* mice with EC-specific expression of human progerin and lamin A together with endogenous mouse lamin A and lamin C [60]. These mice have low body weight, develop cardiac hypertrophy and diastolic dysfunction, and die prematurely at a median age of ≈25 weeks. *Prog-Tg* mice also develop myocardial interstitial and perivascular fibrosis, which was suggested to result from depressed cardiac levels of endothelial nitric oxide synthase (eNOS). Although aortas from *Prog-Tg* mice show no significant VSMC loss or defective acetylcholine-mediated vasorelaxation, they do display adventitial thickening and impaired EC alignment with blood flow. In line with these findings, iPSC-derived ECs from HGPS patients have lower expression of flow-responsive genes than control cells [75]. Moreover, progerin-expressing ECs from *Prog-Tg* mice show increased levels of SUN1 and SUN2, members of the LINC complex, and mislocalization of emerin, a nuclear membrane protein involved in nuclear mechanoresponse [60]. The analysis of *Prog-Tg* mice thus demonstrated a causal role of EC-expressed progerin in the development of some HGPS-associated cardiovascular alterations, suggesting that defective endothelial mechanosensing may be one of the underlying mechanisms. A possible limitation of the *Prog-Tg* model is that ectopic progerin and lamin A are expressed at ≈4 times the level of endogenous lamin A, which could affect the interpretation of the observed alterations.

Another system developed to test the effects of EC-specific progerin expression is the *Lmna^LCS/LCS^Tie2Cre* model [50,51]. These animals were generated by crossing *Lmna^LCS/LCS^* mice [45] with mice expressing Cre recombinase driven by the *Tek* (a.k.a. *Tie2*) promoter [106]. ECs in the progeny *Lmna^LCS/LCS^Tie2Cre* mice express mouse progerin driven by the endogenous *Lmna* promoter. Unlike mice with ubiquitous and *SM22αCre*-driven progerin expression, *Lmna^LCS/LCS^Tie2Cre* mice show no increase in vascular stiffness [51]. Moreover, consistent with the *Prog-Tg* model, *Lmna^LCS/LCS^Tie2Cre* mice lack significant alterations in acetylcholine-mediated vasorelaxation [50]. These studies were therefore unable to demonstrate any effect of EC-specific progerin expression; however, the sacrifice of all animals at 13–15 weeks prevented analysis of survival or any vascular features that may take longer to appear.

A similar *Tie2*-Cre-based strategy was adopted to generate the *Lmna^f/f^*;TC model of EC-specific progerin expression [57]. *Lmna^f/f^*;TC mice share many features with the *Prog-Tg* model, including modest but significant body weight reduction, cardiac hypertrophy, fibrosis and functional alterations, signs of aortic adventitial thickening and reduced eNOS expression, and premature death at a median age of ≈25 weeks. However, unlike *Prog-Tg* and *Lmna^LCS/LCS^Tie2Cre* mice, *Lmna^f/f^*;TC mice also show significantly impaired aortic vasorelaxation in response to acetylcholine, suggesting that progerin expression in ECs may be sufficient to induce endothelial dysfunction. This observation is in line with the impaired vasorelaxation of tissue-engineered blood vessels generated with induced pluripotent stem cell-derived ECs from HGPS donors [75]. The contrasting lack of endothelium-mediated vasodilation defects in *Lmna^LCS/LCS^Tie2Cre* mice [50] could be due to the different ages at which these assays were performed (≈17-week-old *Lmna^f/f^*;TC mice versus ≈14-week-old *Lmna^LCS/LCS^Tie2Cre* mice). Strikingly, aortic atheromatous plaques were found in 100% of analyzed *Lmna^f/f^*;TC mice, a feature not reported in any previous HGPS mouse model with intact *Apoe* and *Ldlr* genes. A possible limitation of the *Lmna^LCS/LCS^Tie2Cre* and *Lmna^f/f^*;TC models is that *Tie2* promoter-driven, Cre-mediated recombination is not strictly limited to ECs and can also occur in certain hematopoietic cell types [107]. Nevertheless, the lifespan of *Lmna^f/f^*;TC mice significantly increased after EC-specific, *Icam2* promoter-driven AAV1-mediated overexpression of SIRT7, a nicotinamide adenine dinucleotide–dependent deacylase [57]. This observation suggests that premature death of *Lmna^f/f^*;TC mice can be partially rescued by targeting ECs. However, it is uncertain whether this strategy would increase lifespan and prevent or correct the cardiac and aortic phenotypes of HGPS mouse models with ubiquitous progerin expression.

The data currently available from various mouse models thus suggest that EC-expressed progerin plays a causal role in the development of structural and functional alterations in cardiac and aortic tissue, which probably contribute to progerin-induced premature death. Since blood is in direct contact with the vascular endothelium, any therapeutic agent reaching the blood stream could potentially access ECs with relative ease. It is therefore tempting to propose EC-targeted therapy as an attractive strategy to counter the HGPS-associated cardiovascular phenotype and eventually prolong lifespan, although much more work is needed to define specific strategies.

## 5. An Integrated Model for the Development of HGPS-Associated Cardiovascular Dysfunction

**Figure 1:**
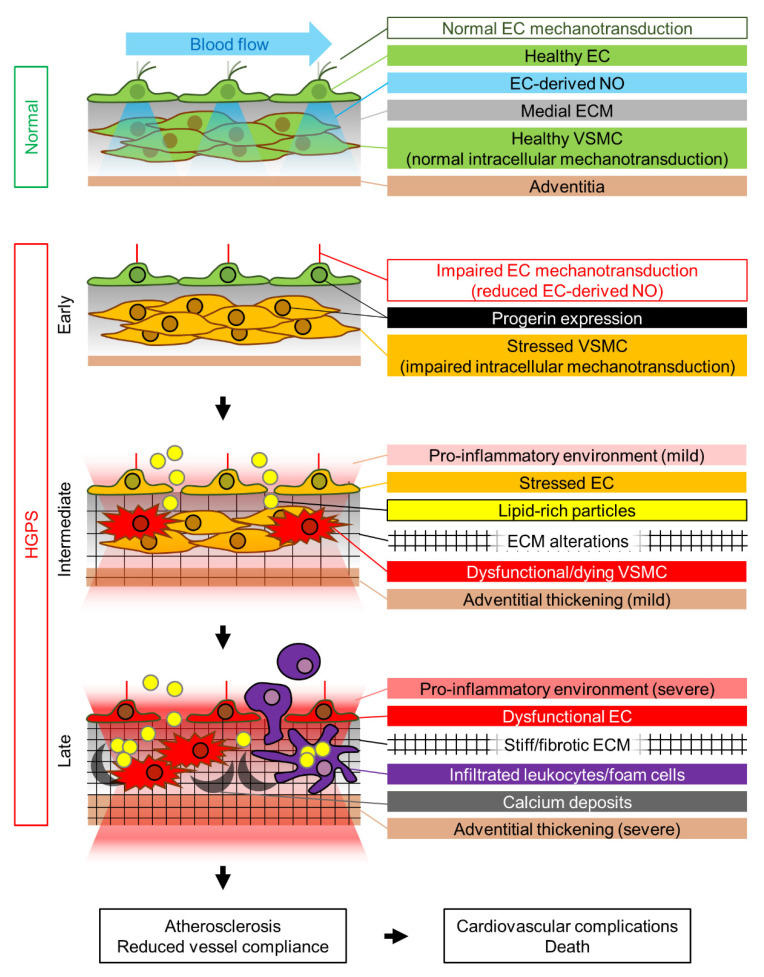
Proposed mechanism of HGPS-associated cardiovascular dysfunction. Progerin expression is represented by a black nuclear rim. EC, endothelial cell; ECM, extracellular matrix; NO, nitric oxide; VSMC, vascular smooth muscle cell.

We propose that the HGPS-associated vascular phenotype is due to progerin-induced alteration of multiple mechanotransduction pathways in the vessel wall that affect both VSMCs and ECs (Figure 1). The pulsatile flow of blood in the arteries subjects the arterial wall to continuous mechanical stress. For reasons that remain elusive, progerin-expressing VSMCs appear to be particularly sensitive to this constant cyclic insult, which induces ER stress, dedifferentiation, calcification (and possibly acquisition of an osteogenic phenotype), DNA damage, and eventually VSMC death, probably due to progerin-mediated altered nucleocytoskeletal connections and defective intracellular force transmission. VSMC damage results in the production of proinflammatory cytokines and ECM remodeling in the arterial adventitial, medial, and probably intimal layers. This proinflammatory environment, together with the alteration of mechanical cues induced by vessel stiffening, likely affects endothelial structure, gene expression, and function. Additionally, progerin-expressing ECs fail to properly mechanotransduce atheroprotective and antifibrotic laminar flow-mediated signals, resulting in decreased eNOS expression. Subsequent reduction in the production of endothelial NO (and probably other angiocrine factors) also contributes to VSMC dedifferentiation and the acquisition of a pro-fibrotic phenotype, thereby establishing a detrimental feedback loop of vascular damage. The reduction in vessel compliance can induce cardiac problems, which may be worsened by the pro-fibrotic environment generated by progerin-expressing cardiac ECs. EC alterations may also further promote atherosclerosis development by increasing endothelial permeability. We propose that the main difference between the mechanisms that regulate atherosclerosis onset in physiological aging and in HGPS is the primary factor inducing endothelial dysfunction. During normal aging, the primary causes of endothelial stress are mainly vessel-extrinsic factors such as dyslipidemia (e.g., hypecholesterolemia, hypertriglyceridemia) and hyperglycemia, which compromise endothelial function and trigger and sustain the vascular damage loop that involves VSMC dysfunction. Conversely, in HGPS patients this cycle is started by VSMC damage, which promotes endothelial dysfunction (Figure 1). Both scenarios ultimately lead to atherosclerosis development and cardiovascular complications, the main cause of death in both the elderly population and HGPS patients.

## 6. Conclusions and Open Questions

Despite the extremely low number of HGPS patients worldwide, several studies over the past decades have defined a characteristic cardiovascular phenotype associated with the disease. Multiple animal models, together with sophisticated in vitro approaches, are starting to unveil the cellular and molecular mechanisms that control the onset and progression of vascular and cardiac alterations induced by progerin expression. Moreover, it is becoming increasingly evident that VSMCs and ECs both play key roles in the acquisition of the HGPS cardiovascular phenotype. Further studies are needed to characterize the micro- and macrovascular endothelium in more detail in different tissues from HGPS animal models. These studies need to address multiple aspects of the endothelial phenotype in situ and assess the gene and protein expression profiles of native, non-cultured ECs. In order to analyze potential causes and outcomes of the HGPS-associated atherosclerotic lesion, these studies should be carried out in both the absence and presence of atherosclerosis, using mice with intact or deleted *Apoe* and/or *Ldlr* genes. It would be also interesting to study the type of plaques present in atheroprone HGPS mouse models and to compare them with those associated with non-HGPS atherosclerosis. The study of plaque features such as lipid content, inflammatory infiltrate, and ECM composition may reveal distinct mechanisms of plaque formation and, more importantly, plaque rupture or erosion, which could help in the design of specific therapeutic approaches for normal versus HGPS-related atherosclerosis. Other important outstanding questions relate to whether progerin-induced vascular damage is reversible and which cell types from the vascular wall and other tissues are the best therapeutic targets for preventing or reversing the HGPS-associated vascular phenotype. This information would aid in the design and development of future therapies aimed at ameliorating progerin-induced cardiovascular alterations, thereby extending the lifespan of HGPS patients.

## Figures and Tables

**Table 1:** Cardiovascular phenotype of HGPS animal models.

			Vascular Features								Cardiac Features					
Species	Animal Model	Progerin Expression	VSMC Loss	Adventitial Thickening/ Fibrosis	Medial Collagen Accumulation	Atherosclerosis	Vascular Stiffening	Altered Vessel Contraction (Phenylephrine)	Altered Vessel Relaxation (Acetylcholine)	Aorta/Carotid Calcification	Coronary/Aortic Root Calcification	Interstitial Fibrosis	Perivascular Fibrosis	Electrical Anomalies	Diastolic Dysfunction	References
*Mus musculus*	*Zmpste24* *^−/−^*	N	NR	NR	NR	NR	NR	NR	NR	NR	NR	Y^qo^/N ^1^	Y	Y	N	[35,38,39]
	G608G BAC	Ubiquitous	Y	Y	Y	NR	NR	NR	NR	Y^ht^	NR	NR	NR	NR	NR	[42,43,44]
	*Lmna^G609G^*		Y	Y	Y	N	Y	Y	Y	Y^ht^	NR	NR	NR	Y	Y/N ^2^	[45,46,47,48,49,50,51,52,53,54,55,56,57]
	*Apoe^−/−^* *Lmna^G609G/G609G^*		Y	Y	NR	Y	NR	NR	NR	NR	N	Y	Y	Y *	NR	[53,58]
	*Ldlr^−/−^* *Lmna^G609G/G609G^*		Y	Y	Y^qo^	Y	NR	NR	NR	NR	NR	NR	Y^qo^	NR	NR	[59]
	*Lmna^LCS/LCS^* *SM22αCre*	VSMCs, cardiomyocytes	Y^qo^	Y^qo^	NR	NR	Y	Y	N	NR	NR	NR	NR	NR	NR	[50,51,53]
	*Apoe^−/−^ Lmna^LCS/LCS^* *SM22αCre*		Y	Y	NR	Y	NR	NR	NR	NR	Y	Y	Y	N *	NR	[53]
	*Lmna^LCS/LCS^* *Tie2Cre*	ECs, some leukocytes	NR	NR	NR	NR	N	N	N	NR	NR	NR	NR	NR	NR	[50,51]
	*Lmna^f/f^;TC*		Y^qo^	Y^qo^	Y^qo^	Y^qo^	NR	NR	Y	NR	NR	Y^qo^	Y^qo^	NR	NR	[57]
	*Prog-Tg*	ECs	N	Y	NR	NR	NR	NR	N	N	NR	Y	Y	NR	Y	[60]
*Sus scrofa*	*LMNA* c.1824C>T Yucatan minipig	Ubiquitous	Y	Y	Y	NR	NR	NR	NR	NR	NR	Y	Y	Y	Y	[61]
*Macaca* *fascicularis*	*LMNA* c.1824C>T cynomolgus monkey		NR	Y	NR	NR	NR	NR	NR	NR	NR	NR	NR	NR	NR	[62]

Y, yes; N, no; NR, not reported; ^ht^, reported only in heterozygous mice; ^qo^, qualitative observation (no quantification). ^1^, Y^qo^ in [39], N in [35,38]. ^2^, Y in [52] (n = 12–16 mice), N in [45] (n = 4 mice). *, results obtained in 16-week-old mice; however, 26-week-old *Apoe^−/−^ Lmna^LCS/LCS^ SM22αCre* mice present modest but significant cardiac electrical anomalies [53].
